# An efficient transition metal chalcogenide sensor for monitoring respiratory alkalosis

**DOI:** 10.1007/s13205-023-03497-z

**Published:** 2023-03-01

**Authors:** Partha Kumbhakar, Mizaj Shabil Sha, Chandra Sekhar Tiwary, Asan G. A. Muthalif, Somaya Al-maadeed, Kishor Kumar Sadasivuni

**Affiliations:** 1 Center for Advanced Materials, Qatar University, PO Box 2713, Doha, Qatar; 2 Indian Institute of Technology, Kharagpur, West Bengal India; 3 Department of Mechanical and Industrial Engineering, Qatar University, PO Box 2713, Doha, Qatar; 4 Department of Computer Science and Engineering, Qatar University, PO Box 2713, Doha, Qatar

**Keywords:** Exhaled breath, Carbon-dioxide, Electrochemical sensors, Respiratory alkalosis

## Abstract

For many biomedical applications, high-precision CO_2_ detection with a rapid response is essential. Due to the superior surface-active characteristics, 2D materials are particularly crucial for electrochemical sensors. The liquid phase exfoliation method of 2D Co_2_Te_3_ production is used to achieve the electrochemical sensing of CO_2_. The Co_2_Te_3_ electrode performs better than other CO_2_ detectors in terms of linearity, low detection limit, and high sensitivity. The outstanding physical characteristics of the electrocatalyst, including its large specific surface area, quick electron transport, and presence of a surface charge, can be credited for its extraordinary electrocatalytic activity. More importantly, the suggested electrochemical sensor has great repeatability, strong stability, and outstanding selectivity. Additionally, the electrochemical sensor based on Co_2_Te_3_ could be used to monitor respiratory alkalosis.

**Supplementary Information:**

The online version contains supplementary material available at 10.1007/s13205-023-03497-z.

## Introduction

Human exhaled breath can be a useful indicator for various issues in our respiratory system and can be used to detect various diseases and conditions. (Sha et al. 2022a, b, c; Sha et al. 2022a, b; Geetha et al. 2022) The body's numerous cellular functions produce CO_2_ as a metabolic byproduct, and it has some systems to control carbon dioxide levels. (Issitt et al. 2022) One is an acid–base buffering mechanism that balances carbon dioxide and bicarbonate levels. Consequently, any changes may lead to imbalance. (Rawat et al. 2022) Respiratory alkalosis results when low carbon dioxide levels disturb the blood’s acid–base equilibrium. People who are fasting or have asthma frequently experience it (hyperventilation). Anxiety or panic attacks, fever, pregnancy, pain, tumors, and trauma are all potential causes of hyperventilation. Any lung condition that causes breathlessness may also result in respiratory alkalosis.

Due to their indirect bandgaps, optoelectronic behavior, and stability, transition metal chalcogenides (TMC) have recently attracted attention from researchers worldwide. (Shen et al. 2022) They are being investigated for use in a variety of applications. Due to their high activity and chemical stability, research on transition metal chalcogenides is expanding for various electrochemical applications, such as batteries, supercapacitors, and low-temperature water electrolysis. (Dasadia and Bhavsar 2022) Their use as electrocatalysts for producing value-added compounds like C-based from the CO_2_ reduction process is particularly intriguing (CO_2_R). Low overpotential, high current density, long-term stability, and economic cost-effectiveness in raw material availability and processing conditions are expected characteristics of an effective and realistically possible electrocatalyst. (Haque et al. 2017; Das et al. 2018, 2019; Shanmugaratnam et al. 2019, 2021; Sajjad et al. 2021)

Co stands out among the transition metals because altering the *d*-electron density near the active site, particularly in the case of a half-filled, e.g., orbital, would result in enhanced charge transferring at the catalyst–water interface. Because of these properties, Co occupies the top position on the Sabatier plot and allows it to exhibit high efficiency for the oxygen evolution reaction (OER) in a basic medium. (Ju et al. 2021) Oxides and hydroxides of cobalt have been carefully examined for OER in basic media as prospective candidates for electrocatalytic water splitting, N–C composites, phosphates, phosphides and metal–organic frameworks explored along with chalcogenides. Tellurides, a group of chalcogenides, have grown in significance in materials chemistry due to the vast range of their diverse characteristics. (Flores-Lasluisa et al. 2022) These tellurides exhibit electron cloud delocalization, improving charge mobility and conductivity. The popularity of nanostructured metal tellurides as catalysts in energy conversion and storage technologies has generated much interest in this field. (Franco et al. 2020).

Two CO_2_ sensors are currently available: electrochemical and non-dispersive infrared (NDIR). Electrochemical sensors measure the CO_2_ concentration by measuring changes in the electrical properties of materials caused by CO_2_ adsorption. They have the advantage of being inexpensive and portable. Recently, transition metal chalcogenides (TMC)-based sensors have emerged a lot. FlorentStarecki et al. developed all-optical carbon dioxide remote sensing using rare earth-doped chalcogenide fibers (Starecki et al. 2019) and IR-emitting Dy3 + doped chalcogenide fibers for in situ CO_2_ monitoring in high-pressure microsystems (Starecki et al. 2016). Frédéric Charpentier et al. developed infrared monitoring of underground CO_2_ storage using chalcogenide glass fibers (Charpentier et al. 2009).

Even though all these work uses chalcogenides for CO_2_ monitoring and sensing, they have some disadvantages, including heavy-duty instruments and are not cost-effective. Herein, we have developed this sensor which can overcome disadvantages with low detection limit, great repeatability, strong stability, and outstanding selectivity.

The electrocatalytic characteristics of cobalt telluride (Co_2_Te_3_) have been discussed in this article. This cobalt telluride exhibits significant catalytic activity for sensing CO_2_ in an alkaline medium (Sha et al. 2022c; Barbee et al. 2022). The cobalt tellurides were fabricated using the liquid phase exfoliation method.

## Materials and methods

### Chemicals

E. Merck provided Cobalt. Sigma Aldrich provided Tellurium powder. Fisher Scientific UK Limited (UK) provided Ethanol and Isopropyl alcohol. Thermo fisher scientific (USA) and AMWAY (India) provided Nafion and sodium hydroxide, respectively. All the chemicals were utilized without further purification. A Millipore-Q deionized (DI) water purification system provided the required water throughout the experiments.

### Synthesis of Co_2_Te_3_

**Fig. 1:**
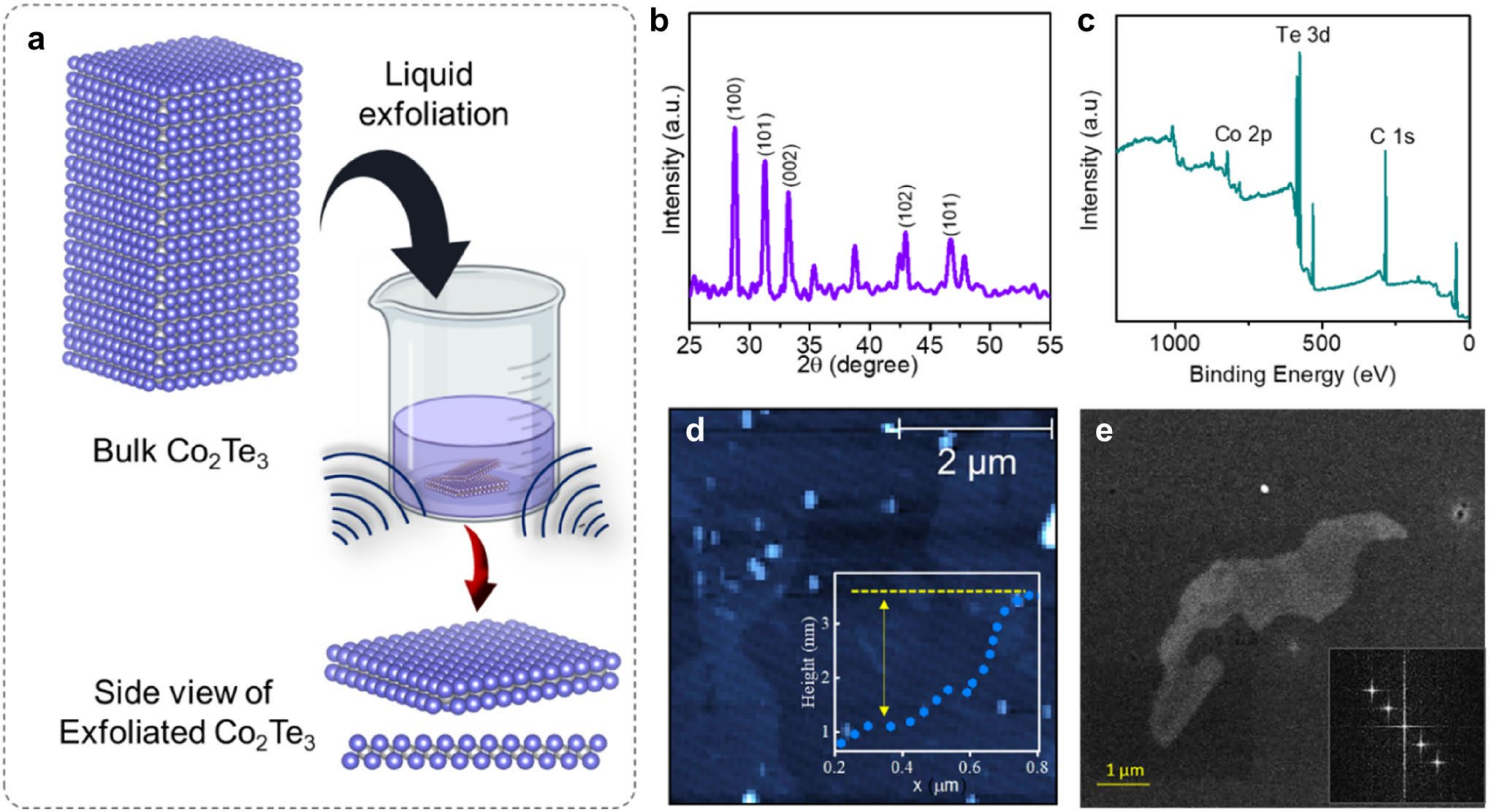
**a** Exfoliation process **b** XRD of 2D Co_2_Te_3_. **c** XPS of the exfoliated sample. **d** AFM images; Inset shows the bar chart presentation of thickness. **e** BF-TEM images of exfoliated Co_2_Te_3_ show 2D sheets

The vacuum induction technique, which entailed melting the component element at 1050 °C in a quartz tube and followed by cooling in an argon environment, was used to create bulk Co_2_Te_3_ material. The Co–Te binary alloy phase diagram was used to identify the sample's stoichiometric makeup. The compound comprising 25 weight percent Co and 75 weight percent Te was made using high-purity components (99.99 percent purity) bought from Sigma Aldrich. This composition range was selected because the Co_2_Te_3_ phase, which contains 60% or more tellurium in the Co–Te system, is more stable. A vacuum pressure of 1 × 10^−5^ mbar was maintained within the melting chamber to achieve extreme purity and avoid oxidation. For further characterization, samples with dimensions 10 × 8 × 1 cm were fabricated, cleaned, and polished. The mechanically ground 50 mg of Co_2_Te_3_ powder was mixed with 150 mL of isopropyl alcohol solvent in an ultrasonic vibrator for 6 h at room temperature to create a suspension of 2D Co_2_Te_3_ nanosheets. This process was called sonication-assisted liquid exfoliation (Fig. 1a). Flakes of few-layer Co_2_Te_3_ were dissolved in the solvent following centrifugal treatment.

### Characterization techniques

Numerous morphological and structural analyses were conducted. An X-ray diffractometer (X'PERT-Pro M.P.D., Netherlands) was used to assess crystallinity. The morphology was investigated using transmission electron microscopy (TEM). Thermo fisher scientific DXR was used for Raman analysis. Atomic force microscopy (AFM) was provided by nanoscience analytical to examine the thin sheets. A detailed microstructural analysis was performed using Thermo Fisher Scientific’s X-ray photoelectron spectroscopy (XPS).

### Electrochemical experiments

Electrochemical experiments were carried out by three electrodes: the counter (graphite rod), working electrode (glassy carbon (diameter 3 mm)), and reference (Ag/AgCl (3 M KCl solution)). A mixture of 20 mg of Co_2_Te_3,_ 2 mL of ethanol, and 20 μL of Nafion was ultrasonicated to produce homogeneous catalytic ink. The working electrode was polished using Aluminum oxide powder and cleaned using distilled water. Afterward, 5 μL of Co_2_Te_3_ suspension was uniformly drop casted on the surface of GCE. and dried under ambient conditions. Electrochemical analyses were performed by Gamry potentiostat/galvanostat (Ref 600) using sodium hydroxide solution as an electrolyte. CO_2_ sensing properties of Co2Te3 were investigated by cyclic voltammetry (CV) experiments with a potential range of − 1.0 to 1.0 V with a 100 mVs^−1^ scan rate in the presence and absence of CO_2_. A 0.1 M NaOH (pH 10) solution was used as an electrolyte.

The repeatability of the Co_2_Te_3_ sensors was analyzed by performing cyclic voltammetry for five different electrodes. For the analysis, the same amount of catalyst was coated on the electrode with the same concentration of CO_2,_ passing in 0.1 M NaOH solution at a scan rate of 100 mV s^−1^. Reproducibility was analyzed by using the same working electrode in five successive runs. The Co_2_Te_3_ electrode exposed stability via cyclic voltammetry for 500 cycles in the aqueous 0.1 N NaOH solution at 100 mVs^−1^. For real-time evaluation, human breath, through blowing, is transferred into the electrochemical cell containing 0.1 N pH10 NaOH solution. Cyclic voltammetry (CV) experiments were carried out by sweeping potential between − 1.0 and 1.0 V with a scan rate of 100 mVs^−1^.

## Results and discussion

### Characterization of Co_2_Te_3_

Structural characterization and composition of Co_2_Te_3_ samples were examined by X-ray diffraction (XRD), XPS spectra, Raman spectra and elemental mapping. The corresponding diffractogram associated with the material is shown in Fig. 1b, indicating a crystalline phase in the sample. (Negedu et al. 2021) Peaks of (100), (101), (002), (102), (110), and (103) were observed in the 2D Co_2_Te_3_ (Fig. 1b). The (100), (101) and (002) diffraction peaks are the most prominent of these peaks, which suggests that these planes were exfoliated in excess during the exfoliation method compared to other planes from bulk samples. The different diffraction peaks at (100) for 2D and (101) for bulk show the preferential plane orientation of each sample.

Detailed microstructural analysis using the XPS analysis characterized the chemical states and surface elemental composition of Co_2_Te_3_ samples (Fig. 1c). The elemental analysis by EDX Spectroscopy revealed that the material was made entirely of Co and Te elements having identical atomic ratios of Te and Co, as predicted (Supplementary Fig. 1).

AFM studies confirmed the formation of 2D nanosheets and revealed a thin sheet of a thickness of ~ 2 nm, as shown in Fig. 1d and its inset. Bright-field (BF) TEM measurements were used to study the morphology of 2D Co_2_Te_3_, which revealed ultrathin flakes with 200–500 nm (Fig. 1e). The BF-TEM image presents the materials’ structure and changes in the surface morphology after exfoliation. Such sheet-like structures have large surfaces, significantly improving electrochemical sensing properties. The Raman spectra (Supplementary Fig. 2) revealed the main characteristic peak for A_1g_ and E_2g_ symmetry. The Raman peaks of exfoliated Co_2_Te_3_ at 53, 180, 320, and 377 cm^−1^ are compatible with the in-plane and out-of-plane vibrational modes of transition metal chalcogenides (TMC) (Negedu et al. 2021).

### Electrochemical analysis

#### Detection of CO_2_

**Fig. 2:**
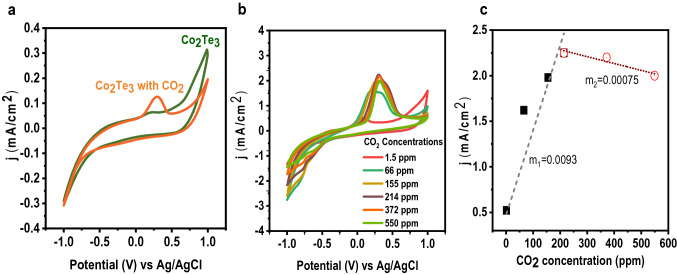
**a** Cyclic voltammograms of Co_2_Te_3_ electrode with and without CO_2_ gas **b** Cyclic voltammograms of Co_2_Te_3_ with different concentrations of CO_2_. **c** A linear relationship between current and the concentration of CO_2_

CO_2_ sensing properties of Co_2_Te_3_ were investigated by cyclic voltammetry (CV) (Fig. 2a). In the absence of CO_2_, no oxidation peak was observed. The new peak was attributed to the NaOH solution’s electrochemical experiment after adding CO_2_ at ~ 0.28 V (Fig. 2a). The CV graphs collected in the presence of different CO_2_ concentrations produced an oxidation peak of 0.2–0.5 V. (Fig. 2b). In the current investigation, CO_2_ gas was employed under controlled cylinder monitoring for the sensing measurement. We employed the straightforward titration approach to ascertain the quantity/concentration of CO_2_ dissolved in the electrolyte. The concentration of CO_2_ in the solution was determined by altering the color of the solution using a phenolphthalein indicator. After that, we used the law of chemical equivalence formula,

1 $$N_{1} V_{1} = N_{2} V_{2} ,$$

where *V*_1_ = volume of the solution, *V*_2_ = volume of NaOH, *N*_1_ is the normality of the solution, and *N*_2_ = normality of Base, here NaOH. The amount of CO2 content in the solution is *N*_1_* the equivalent weight of CO2 in gm/L. Oxidation of adsorbed hydroxyl species takes place at 1.0 V. After adding CO_2_ to the Co_2_Te_3_ electrode, a little change in oxidation current is observed. Results were achieved in 0.1 M NaOH at 0.3 V potential with increasing CO_2_ concentration (1.5–550 ppm). The Co_2_Te_3_ electrode exhibited a rapid response when CO_2_ was introduced, and the concentration of CO_2_ was less than 200 ppm. A linear response with concentration was observed, which signifies the sensing capabilities of Co_2_Te_3_ in the presence of CO_2_. The linear correlation between CO_2_ concentration and the current is displayed in Fig. 2c. The respective slopes were 9.3 × 10^−3^ and 7.5 × 10^−4^, at a lower (1.5–200 ppm) and higher concentration (200–550 ppm), respectively.

#### Effect of scan rate

**Fig. 3:**
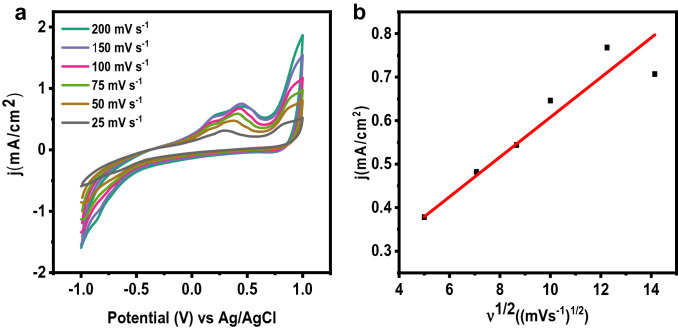
**a** Cyclic voltammograms of CO_2_ sensing at different scan rates **b** Line fitting of oxidation current vs. square root of scan rate

In addition, CV experiments for CO_2_ sensing were carried out at different scan rates (25, 50, 75, 100, 150, and 200 mV s^−1^), and the oxidation peak currents were plotted against the square root of scan rates (Fig. 3). Concerning the increase in scan rate, an increase in the anode peak and a decrease in the cathodic peak have also been noted. Here it should be highlighted that a relationship between the anode/cathode peak magnitudes and the square root of the scanning rate sheds light on the reaction process. The linear regression equation of oxidation peak current for detecting CO_2_ is *j* (mA cm^−2^) = 0.04574*x* + 0.150 (*R*^2^ = 0.892), where x stands for the square root of scan rate, *ν*^1/2^ (Fig. 3b). The graph supports the linear connection regardless of the scan rate. This demonstrates that the ion’s diffusion rate regulated the decrease of CO_2_ to the active sites (Sha et al. 2022c).

#### Selectivity, repeatability, reproducibility and stability

**Fig. 4:**
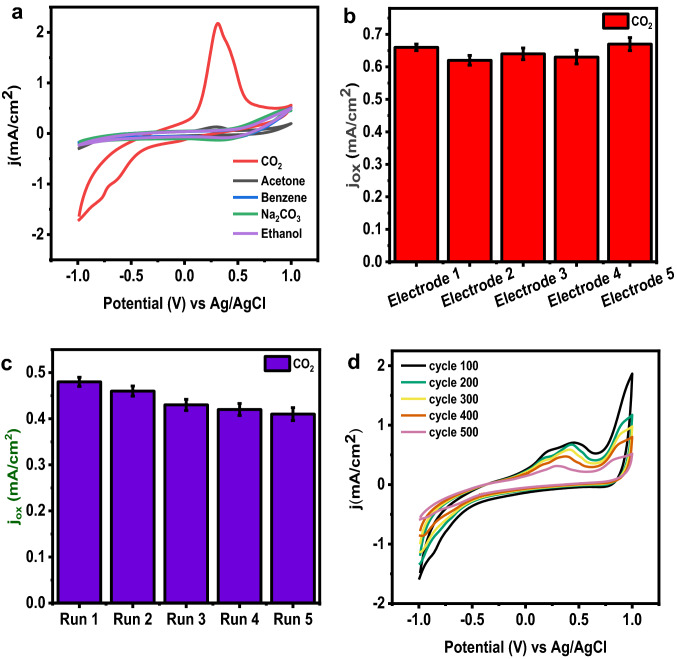
**a** Selectivity analysis using other interference **b** Repeatability using five different working electrodes **c** Reproducibility using the same working electrode conditions **d** Stability analysis

A biosensor must distinguish between electroactive interfering species and other species. The choice of certain species is crucial in most investigations. Therefore, we have used other interfering chemicals, such as acetone, ethanol, benzene, and Na_2_CO_3_. for selectivity analysis. Methanol and ethanol are well recognized to have the potential to interfere with the conductometric detection of CO_2_. But unlike ethanol, which is only found in significant amounts in those who drink alcohol, methanol—a hazardous chemical for humans—is not found in the breath. Therefore, they are absent in humans' breath under normal physiological conditions and do not alter CO_2_ signals. The presence of CO_2_ increases the oxidation current immediately and significantly. Other samples show no such oxidation current, as shown in Fig. 4a. Therefore, the Co_2_Te_3_ catalyst exhibits exceptional selectivity for CO_2_ oxidation.

A sensor should have excellent repeatability, reproducibility and durability. The repeatability analysis (Fig. 4b) revealed a consistent current response across all studies, confirming the sensor’s excellent reproducibility. The analysis observed that the newly designed transition metal chalcogenides (TMC) could act as an efficient electrochemical sensor.

Figure 4c shows that the five tests’ electrochemical responses show the sensor’s excellent repeatability. The electrochemical response decreases slightly with the same working electrode under a similar environment (the same concentration of CO_2_ passing in 0.1 M NaOH solution at a scan rate of 50 mV s^−1^). This could be due to the reduction in the number of active sites in the transition metal chalcogenides (TMC) after each run.

Figure 4d represents the stability analysis. Interestingly CO_2_ revealed higher retention of the current throughout the experiment, demonstrating greater durability/stability. This process can be linked to the oxidation/reduction-induced creation of new active sites.

### Real-time evaluation of the sensor

**Fig. 5:**
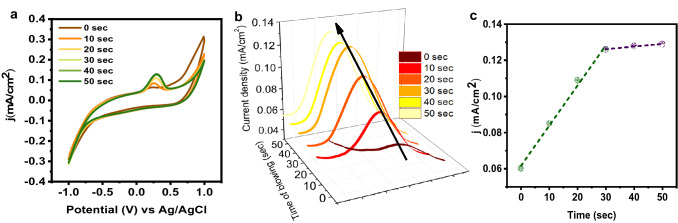
**a** CV graphs for different concentrations of CO_2_ **b** Increase of current with increasing concentration of CO_2_ **c** Linear response in CO_2_ concentration of human breath

To confirm the reliability of the sample for detecting CO_2_ in real examples, this Co_2_Te_3_ electrode was utilized to measure the presence of CO_2_ in human breath. Since human blood samples from diabetes patients have much higher CO_2_ concentrations than usual, electrochemical experiments were carried out by incorporating exhaled breath. Figure 5 suggests that Co_2_Te_3_ electrodes have a dependable anti-interference trait and may be applied in real biotechnological applications. As blowing time increases, a slight increase in current density is observed (Figs. 5b). The obtained analytical results are displayed in Fig. 5b, c. Figure 5c represents the linear relationship between the oxidation current and the time to blow CO_2_ to the cell. It was observed that, till 30 s, there was a rapid increase in current density, whereas afterward, a slight change was observed. Two regions were observed, up to 30 s, (*y* = 2.22 × 10^−3^*x* + 0.0617, *R*^2^ = 0.98924) and from 30 to 50 s (*y* = 0.12*x* + 1 × 10^−4^, *R*^2^ = 0.92857). The excellent linear response of CO_2_ illustrated that the reported method could be utilized effectually to determine CO_2_ in real human breathing.

## Conclusions

In conclusion, our study illustrated the outstanding and adaptable electrochemical sensing capabilities of novel electrodes based on Co_2_Te_3_ for creating high-performance biological sensors. A facile, easily controllable, inexpensive liquid phase exfoliation synthetic method was used to fabricate two-dimensional transition metal chalcogenides. XRD, RAMAN, TEM, EDX, XPS, and AFM characterized the transition metal chalcogenide. This transition metal chalcogenide (TMC) is highly efficient in the electrochemical sensing of carbon dioxide. Additionally, it was observed that this sensor exhibits excellent selectivity, repeatability, reproducibility and stability. These preliminary findings were used to monitor breath carbon dioxide in human test participants to evaluate their practical application. These findings also provide a fresh approach to creating an environment-friendly chemical sensor that employs an environment-friendly chemical sensor for monitoring respiratory alkalosis.

## Supplementary Information

Below is the link to the electronic supplementary material.

Supplementary file1 (DOCX 130 KB)

## Data Availability

The data supporting this study's findings are available at the request of the corresponding author.
