# Supplementary material for: An efficient transition metal chalcogenide sensor for monitoring respiratory alkalosis
Source: 3 Biotech. 2023 Mar 1;13(3):109. doi: 10.1007/s13205-023-03497-z (PMC9978044; doi:10.1007/s13205-023-03497-z)
Supplement: Supplementary file 1 — Supplementary file1 (DOCX 130 KB) [file 13205_2023_3497_MOESM1_ESM.docx]

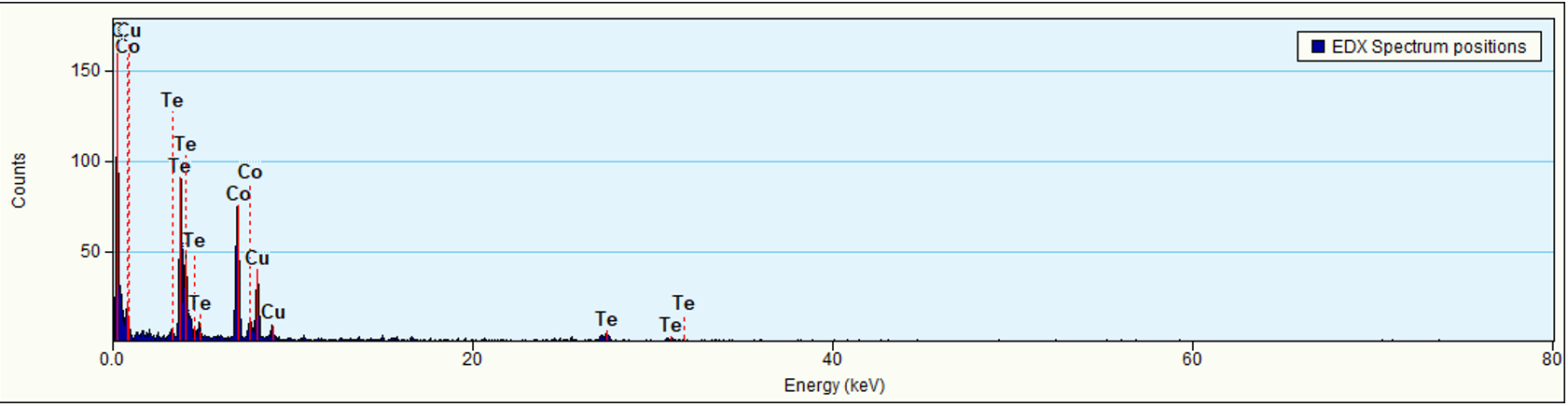


**Supplementary Figure 1.** EDX pattern of exfoliated Co2Te3


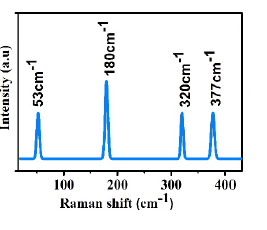


**Supplementary Figure 2.** Raman analysis of the synthesized Co2Te3 measured at room temperature under excitation of λ = 532 nm.
